# Pseudane-VII Isolated from *Pseudoalteromonas* sp. M2 Ameliorates LPS-Induced Inflammatory Response In Vitro and In Vivo

**DOI:** 10.3390/md15110336

**Published:** 2017-11-01

**Authors:** Mi Eun Kim, Inae Jung, Jong Suk Lee, Ju Yong Na, Woo Jung Kim, Young-Ok Kim, Yong-Duk Park, Jun Sik Lee

**Affiliations:** 1 Department of Life Science, Immunology Research Lab, BK21-plus Research Team for Bioactive Control Technology, College of Natural Sciences, Chosun University, Dong-gu, Gwangju 61452, Korea; kimme0303@naver.com (M.E.K.); inae673@naver.com (I.J.); skwndyd@naver.com (J.Y.N.); 2 Biocenter, Gyeonggido Business & Science Accelerator (GBSA), Suwon, Gyeonggi-do 16229, Korea; leejs@gbsa.or.kr (J.S.L.); whehdcks@gmail.com (W.J.K.); 3 Biotechnology Research Division, National Institute of Fisheries Science (NIFS), Gijang, Busan 46083, Korea; nasungong@naver.com; 4 Djkunghee Hospital, Department of Preventive and Society Dentistry, School of Dentistry, Kyung Hee University, Seoul 02447, Korea; iam2875@knu.ac.kr

**Keywords:** inflammation, pseudane-VII, iNOS, MAPK, anti-inflammatory therapeutics

## Abstract

The ocean is a rich resource of flora, fauna, food, and biological products. We found a wild-type bacterial strain, *Pseudoalteromonas* sp. M2, from marine water and isolated various secondary metabolites. Pseudane-VII is a compound isolated from the *Pseudoalteromonas* sp. M2 metabolite that possesses anti-melanogenic activity. Inflammation is a response of the innate immune system to microbial infections. Macrophages have a critical role in fighting microbial infections and inflammation. Recent studies reported that various compounds derived from natural products can regulate immune responses including inflammation. However, the anti-inflammatory effects and mechanism of pseudane-VII in macrophages are still unknown. In this study, we investigated the anti-inflammatory effects of pseudane-VII. In present study, lipopolysaccharide (LPS)-induced nitric oxide (NO) production was significantly decreased by pseudane-VII treatment at 6 μM. Moreover, pseudane-VII treatment dose-dependently reduced mRNA levels of pro-inflammatory cytokines including *inos*, *cox-2*, *il-1β*, *tnf-α*, and *il-6* in LPS-stimulated macrophages. Pseudane-VII also diminished iNOS protein levels and IL-1β secretion. In addition, Pseudane-VII elicited anti-inflammatory effects by inhibiting ERK, JNK, p38, and nuclear factor (NF)-κB-p65 phosphorylation. Consistently, pseudane-VII was also shown to inhibit the LPS-stimulated release of IL-1β and expression of iNOS in mice. These results suggest that pseudane-VII exerted anti-inflammatory effects on LPS-stimulated macrophage activation via inhibition of ERK, JNK, p38 MAPK phosphorylation, and pro-inflammatory gene expression. These findings may provide new approaches in the effort to develop anti-inflammatory therapeutics.

## 1. Introduction

Inflammation is a major component of the innate immune response to harmful stimuli, such as pathogen infection, and is related to many pathophysiological processes [1,2]. In particular, macrophages stimulated by inflammatory stimuli produce pro-inflammatory factors, such as cytokines and nitric oxide (NO) [3]. NO has a critical role in many diseases, including diabetes, rheumatoid arthritis, and atherosclerosis [4,5,6]. NO is produced by inducible NO synthase (iNOS) in macrophages under various inflammatory conditions that is up-regulated by inflammatory stimuli including lipopolysaccharide (LPS), and it can lead to excessive NO production by activated macrophages [7,8]. LPS stimulates toll-like receptor-4 (TLR-4), which triggers the activation of mitogen-activated protein kinase (MAPK) [9]. Activated MAPK is an important activity signal transducer for iNOS and other mediators of cellular responses and pro-inflammatory cytokine expression, especially interleukin (IL)-1β, IL-6, and tumor necrosis factor (TNF)-α [10,11,12]. Therefore, reducing NO and pro-inflammatory cytokine production has been suggested as an effective strategy for suppressing inflammation.

*Pseudoalteromonas* is a genus of marine bacteria and a genus of gram negative marine bacteria. It can be found in open ocean sea water or coastline sea water and organelles consist of curved rods and a single polar flagellum. *Pseudoalteromonas* sp. M2 is a new genus originated from *Alteromonas* [13]. *Pseudoalteromonas* species are commonly found in association with eukarytotic hosts in the marine environment and produce biologically active metabolites.

Pseudane-VII is a novel compound that is a secondary metabolite of *Pseudoalteromonas* sp. M2 and is a member of 4-hydroxy-2-alkylquinolines isolated from natural sources. We recently reported that liquid chromatography–mass spectrometry-based rapid secondary-metabolite profiling of marine *Pseudoalteromonas* sp. M2, and isolated various pseudane compounds as secondary-metabolites, and found pseudane compounds has anti-melanogenic effect [14].

Until now, the mechanism of pseudane-VII anti-inflammatory action on macrophages has not been described.

This study was performed to elucidate the anti-inflammatory effects of pseudane-VII on LPS-stimulated macrophages and to examine the potential mechanisms by which pseudane-VII regulates NO production. We also investigated whether pseudane-VII could regulate the expression of pro-inflammatory cytokines including IL-1β, TNF-α and IL-6 in LPS-activated macrophages. Pseudane-VII reduced LPS-induced NO production and pro-inflammatory cytokine expression in macrophages by decreasing ERK, JNK, and p38 MAPK phosphorylation.

## 2. Results

### 2.1. LC-MS Analysis of the Secondary Metabolite

**Figure 1:**
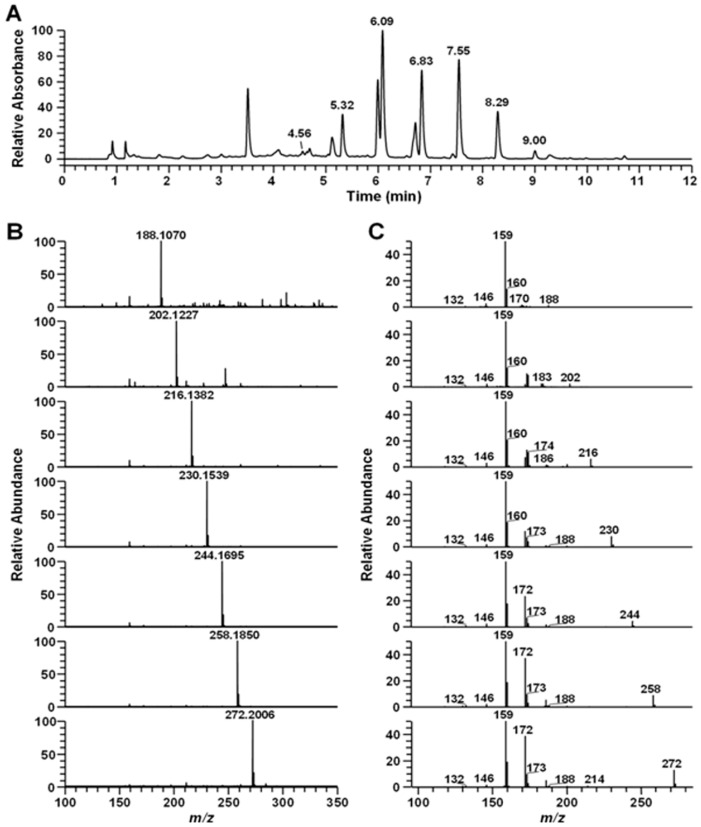
LC-HR-MS/MS analysis of the ethyl acetate extract from *Pseudoalteromonas* sp. M2. culture supernatant: (**A**) PDA chromatogram of ethyl acetate extract; (**B**) high-resolution mass spectrum; and (**C**) MS/MS spectrum of seven-pseudane series (pseudane-III (*m*/*z* 118.1070), pseudane-IV (*m*/*z* 202.1227), pseudane-V (*m*/*z* 216.1382), pseudane-VI (*m*/*z* 230.1539), pseudane-VII (*m*/*z* 244.1695), pseudane-VIII (*m*/*z* 258.1850), and pseudane-IX (*m*/*z* 272.2006)).

The structure of the secondary metabolite from *Pseudoalteromonas* sp. M2 culture extract is shown in Figure 1. The high-resolution mass and MS/MS spectral characteristics of the 4-quinolones were compared to commercially obtained standards and published data.

The main compound was identified as pseudane-VII by AntiBase database search and confirmed by comparison analysis with a standard. The others peaks were detected before and after the major peaks (Figure 1). The high-resolution mass spectrum showed an *m*/*z* 188.1070, 202.1227, 216.1382, 230.1539, 244.1695, 258.1850 and 272.2006 ([M + H]^+^), which was tentatively identified as pseudane-III to IX based on the high-resolution mass and MS/MS production ions, respectively. Thus, the LC-MS/MS analysis of the *Pseudoalteromonas* sp. M2 strains identified seven secondary metabolite peaks as known or putative structures, including pseudane-III (4.56 min) pseudane-IV (5.32 min), pseudane-V (6.09 min), pseudane-VI (6.83 min), pseudane-VII (7.55 min), pseudane-VIII (8.29 min) and pseudane-IX (9.00 min) were determined as a secondary metabolites.

### 2.2. Purification and Analysis of Pseudane VII

**Figure 2:**
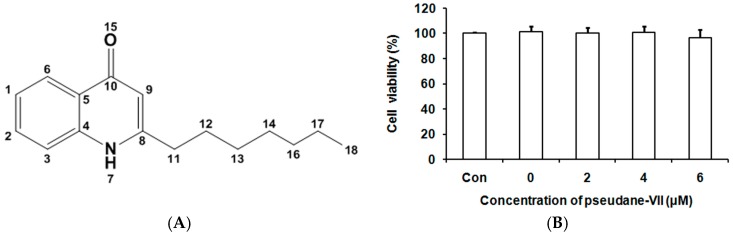
Pseudane-VII was not cytotoxic to RAW 264.7 macrophages. RAW 264.7 cells were seeded into 96-well cell culture plates. Structure of pseudane-VII (**A**). Various concentrations of pseudane-VII (0–6 μM) and vehicle control (0.1% DMSO) were added, and the numbers of live cells were assessed by MTT assay after 24 h, as described in the Materials and Methods. Data are reported as live cell numbers expressed as a percentage of vehicle control cells (**B**). The data represent the average (±SD) of four replicate wells in three separate experiments.

To obtain secondary metabolites, 16 L of cell culture medium was centrifuged and extracted using ethyl acetate. Pseudane-IV, -V, -VI, -VII, -VIII and -IX were purified using an AutoPurification system (Waters, Milford, MA, USA), and we obtained 3.07, 14.96, 10.21, 11.37, 3.18 and 1.10 mg, respectively. The purified pseudane VII (Figure 2A) samples were used in bioactivity assays and nuclear magnetic resonance (NMR) structure analyses (Table 1). The ^1^H NMR analysis of pseudane VII chemical shifts (400 MHz, MeOD) were: δ ppm 0.87 (t, *J* = 6.77 Hz, 3H) 1.10 (quin, *J* = 7.54, 7.40 Hz, 2H) 1.19 (quin, *J* = 7.40 Hz, 2H) 1.28 (tq, *J* = 7.40, 6.77 Hz, 2H) 1.28 (tt, *J* = 7.54, 6.60 Hz, 2H) 1.72 (tt, *J* = 7.50, 6.60 Hz, 2H) 2.68 (t, *J* = 7.50 Hz, 2H) 6.24 (s, 1H) 7.33 (t, *J* = 8.05, 7.61 Hz, 1H) 7.58 (t, *J* =7.96, 7.61 Hz, 1H) 7.71 (d, *J* = 7.96 Hz, 1H) 8.36 (d, *J* = 8.05 Hz, 1H).

### 2.3. Pseudane-VII Inhibited NO Production in LPS-Stimulated Macrophages

**Figure 3:**
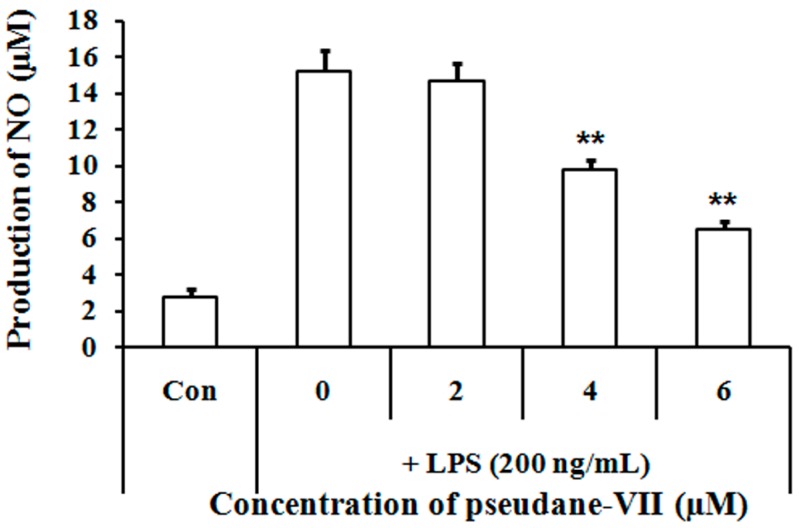
Pseudane-VII decreased LPS-induced NO production in RAW 264.7 macrophages. RAW 264.7 cells were seeded into 96-well cell culture plates. Various concentrations of pseudane-VII (0–6 μM) were used to pre-treat cells for 2 h, and then the cells were incubated for 24 h in the presence or absence of LPS (200 ng/mL). Supernatants were mixed with Griess reagent, and absorbance was measured at 540 nm on a microplate reader. The data represent the average (±SD) of four replicate wells and are representative of three separate experiments. (** *p* < 0.01 vs. LPS only groups).

We first examined the cytotoxicity of pseudane-VII in a macrophage cell line using colorimetric MTT assay. Cells were cultured in the presence of various concentrations of pseudane-VII (2, 4 and 6 μM) for 24 h, and no cytotoxicity was observed (Figure 2B). To investigate whether pseudane-VII could regulate NO production, we measured NO levels in LPS-stimulated macrophages after pseudane-VII treatment. Macrophages were pre-treated with pseudane-VII for 2 h and then stimulated with LPS for 24 h. LPS treatment significantly induced NO production compared with control (Figure 3). However, pre-treatment of macrophages with pseudane-VII significantly inhibited LPS-induced NO production in a dose-dependent manner. These results show that pseudane-VII inhibited NO production in LPS-stimulated macrophages at non-cytotoxic concentrations.

### 2.4. Pseudane-VII Suppressed Pro-Inflammatory Enzyme Expression

**Figure 4:**
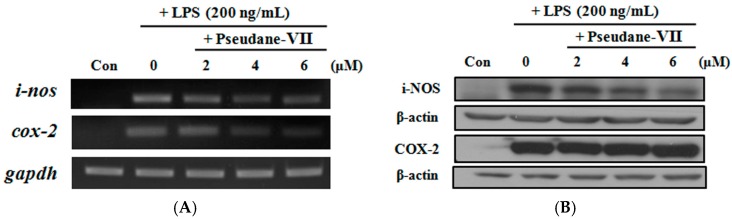
Pseudane-VII suppressed LPS-induced pro-inflammatory enzymes in RAW 264.7 macrophages. The *inos* and *cox-2* mRNA and protein expression were determined by: RT-PCR (**A**); and Western blot analysis (**B**). RAW 264.7 cells were pre-treated with various concentrations of pseudane-VII (0–6 μM) for 2 h and stimulated with LPS (200 ng/mL) for 6 h (RT-PCR) or 24 h (Western blot). Pseudane-VII treatment reduced *inos* and *cox-2* mRNA levels, as well as iNOS and COX-2 protein level. The respective internal controls were *gapdh* and β-actin. The data are representative of four separate experiments.

iNOS and COX-2 are the principle inflammatory enzymes and produce NO and prostaglandin E2, which are mediators under the inflammatory conditions [14]. To examine the effects of pseudane-VII on *inos* and *cox-2* mRNA expression, we performed reverse-transcriptase polymerase chain reaction (RT-PCR) assays. Macrophages were pre-treated with or without pseudane-VII for 2 h and then stimulated with LPS for 6 h. LPS stimulation increased mRNA expression levels of *inos* and *cox-2*, which were significantly reduced by pseudane-VII in a dose-dependent manner (Figure 4A). In addition, we measured the protein expression levels of iNOS and COX-2 by Western blot analysis. Protein expression level of iNOS and COX-2 were induced by LPS stimulation. Pseudane-VII treatment suppressed the protein expression of iNOS but not COX-2 (Figure 4B). These results indicate that pseudane-VII reduced NO production by inhibiting iNOS expression.

### 2.5. Pseudane-VII Diminished Pro-Inflammatory Cytokine Production

**Figure 5:**
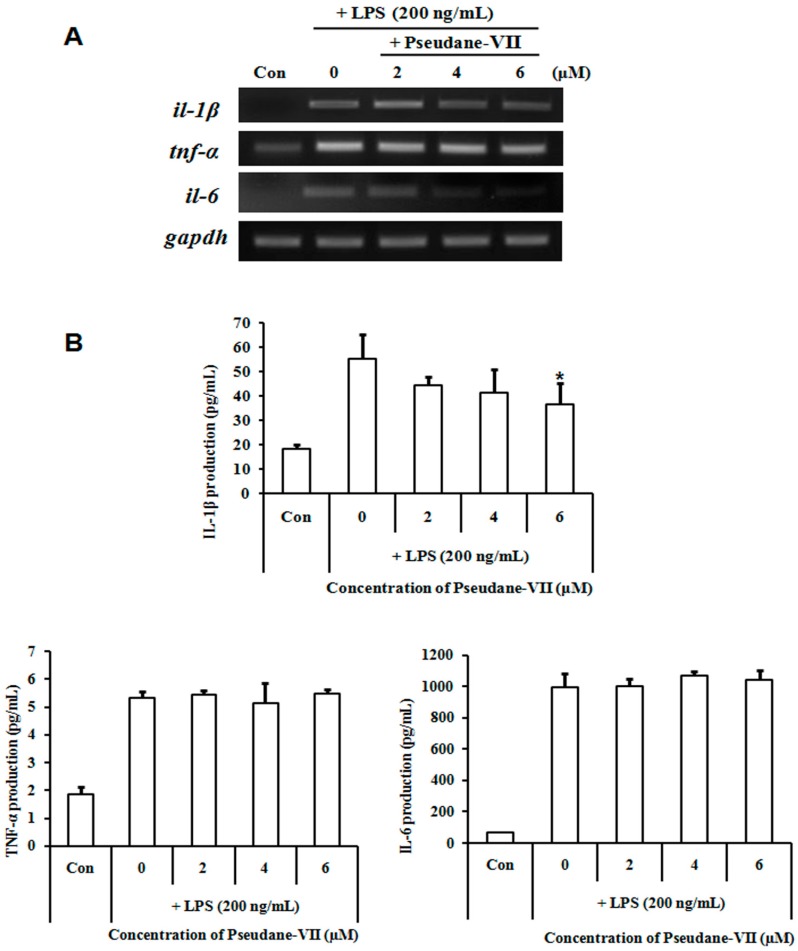
Pseudane-VII suppressed LPS-induced cytokine production. Pro-inflammatory cytokine (IL-1β, IL-6 and TNF-α) levels were examined by: RT-PCR (**A**); and ELISA (**B**). RAW 264.7 cells were pre-treated with pseudane-VII (0–6 μM) followed by LPS (200 ng/mL) stimulation for 6 h (RT-PCR) or 24 h (ELISA). Pseudane-VII treatment diminished LPS-induced pro-inflammatory cytokine mRNA levels and secretion. The data represent the average (±SD) of four replicate wells and are representative of three separate experiments. (** *p* < 0.01 vs. LPS only groups).

Macrophages generally produce pro-inflammatory cytokines such as IL-1β, IL-6 and TNF-α under various inflammatory conditions. We performed RT-PCR to determine the effects of pseudane-VII on *il-1β*, *il-6*, and *tnf-α* mRNA expression. Macrophages were pre-treated with or without pseudane-VII for 2 h followed by LPS activation for 6 h. The mRNA expressions of *il-1β* and *il-6* were increased by LPS treatment, but pseudane-VII markedly reduced mRNA levels (Figure 5A). To measure the production of pro-inflammatory cytokines including IL-1β, IL-6, and TNF-α by enzyme-linked immunosorbent assay (ELISA), macrophages were pre-treated with pseudane-VII for 2 h followed by LPS treatment for 24 h. LPS-activated macrophages increased the secretion of IL-1β, IL-6 and TNF-α, but IL-1β production was significantly and dose-dependently reduced by pseudane-VII (Figure 5B). These results showed that pseudane-VII selectively inhibited the expression and production levels of IL-1β.

### 2.6. Pseudane-VII Inhibited LPS-Induced MAPK Activation and NF-κB Signaling

**Figure 6:**
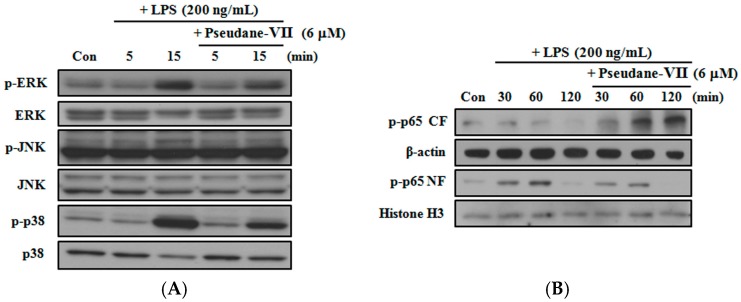
Pseudane-VII inhibited LPS-induced MAPK phosphorylation and NF-κB translocation. RAW 264.7 cells were starved in serum-free DMEM and pre-treated with 6 μM pseudane-VII for 2 h. Cells were then stimulated with 200 ng/mL LPS for indicated times. Total cell lysates were used for MAPK detection (**A**), and nuclear fraction/cytosolic fractions were used to detect the NF-κB-p65 subunit (CF: Cytosol Fraction; and NF: Nucleus Fraction) (**B**). Equal amounts of proteins were separated by SDS-PAGE and transferred to PVDF membranes that were probed with antibodies. β-actin and histone-H3 were used as internal controls. The results are from one experiment representative of four performed that showed similar patterns.

MAPKs such as ERK1/2, p38 and JNK are generally activated by TLR4 receptor and sequentially trigger nuclear factor (NF)-κB phosphorylation and translocation in some mammalian cells [15,16]. To verify the molecular mechanism of the anti-inflammatory activity of pseudane-VII on RAW 264.7 macrophages, we determined the phosphorylation status of MAPKs and NF-κB in LPS-activated macrophages. Phosphorylation of ERK1/2, p38 and JNK increased after 5 and 15 min in LPS-induced macrophages. Treatment with pseudane-VII significantly inhibited the LPS-induced phosphorylation of p38 and ERK1/2 (Figure 6A). Moreover, LPS activation also induced NF-κB translocation from the cytosol to the nucleus, and this was inhibited by pseudane-VII treatment (Figure 6B). The results indicate that pseudane-VII inhibits pro-inflammatory factors via regulation of MAPKs and NF-κB signaling in LPS-stimulated macrophages.

### 2.7. Pseudane-VII Decreased iNOS Expression and IL-1β Production in LPS-Treated Mice

**Figure 7:**
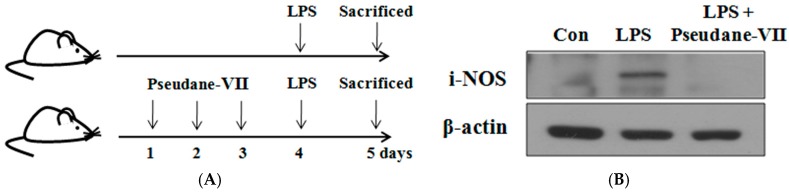

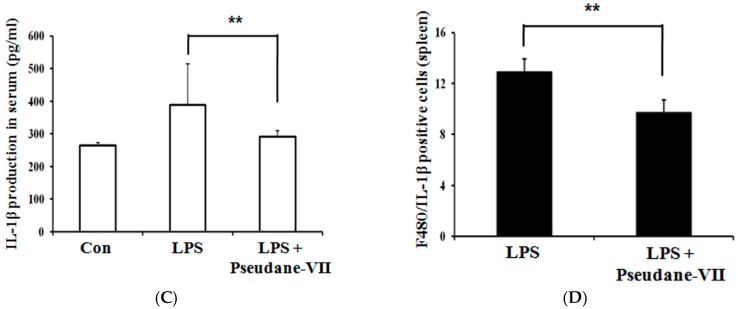
Pseudane-VII regulates inflammatory response in LPS-treated mice. Scheme of the in vivo study design (**A**). C57BL/6 mice were randomly divided into three groups. The control group was challenged the same amount of solvent i.p. (*n* = 5). The treatment group was administered LPS (1 mg/kg) and pseudane-VII (1 mg/kg) as followed in vivo study design. Twenty-four hours after the injection, blood and spleen were harvested. From the spleen, prepared protein lysates were used for iNOS detection (**B**). The serum was separated to measure IL-1β levels by ELISA (**C**). Splenocytes were stained with F480-FITC (for macrophages) and IL-1β-PE antibodies, and was measured by flow cytometry (**D**). The results are from one experiment representative of four performed that showed similar patterns. The data represent the average (±SD) of four replicate wells and are representative of three separate experiments. (** *p* < 0.01 vs. LPS only groups).

LPS has been investigated as an agent for mimicking the initial clinical features of systemic inflammation [17]. For this reason, we determined the therapeutic efficacy of pseudane-VII in LPS-treated mice. As shown in Figure 7, the LPS-stimulated production levels of IL-1β in serum and iNOS in spleen were significantly induced when compared to the control mice group. When treated with 1 mg/kg of pseudane-VII, levels of IL-1β production and iNOS expression were dramatically suppressed. Moreover, we tried to determine the IL-1β-secreting macrophages populations of LPS-treated mice group compared with pseudane-VII plus LPS-treated mice group. Splenic macrophage of LPS-treated group increased IL-1β expression, but these changes were significantly reduced by pseudane-VII treatment (Figure 7D).

## 3. Discussion

Inflammation is a primary response of the innate immune system against exogenous infections such as bacteria. Macrophages are a major immune cell type that has critical roles in inflammation and tissue repair [18,19,20]. However, excessive macrophage activation in local sites causes chronic inflammatory diseases such as fibrosis, diabetes, atherosclerosis, and cancers [21,22,23,24]. The results of this study demonstrate that pseudane-VII, a novel compound from *Pseudoalteromonas* sp. M2 metabolites, inhibits the expression of pro-inflammatory mediators such as iNOS, IL-6, and IL-1β via suppression of LPS-activated MAPK phosphorylation and NF-κB translocation. Therefore, our study provides novel insight into the possible mechanisms underlying the anti-inflammatory activities of pseudane-VII. 

NO is a reactive molecule that acts as a powerful inflammatory indicator in biological systems. NO can be produced by iNOS expression and is generated by activated macrophages in inflammatory conditions. This physiologic double-edged sword has preventive activities against exogenous pathogens, but excessive NO production has been related to various chronic inflammatory diseases [25].

Inflammation is attributed to the activation of pro-inflammatory cytokines including IL-1β, TNF-α and IL-6, which contribute to the regulation of inflammation, immune responses, and tissue repair through their effects on NO production and autocrine/paracrine activities in immune cells [26,27]. For that reason, inhibition of NO and pro-inflammatory cytokine production by macrophages is considered a therapeutic strategy for the development of anti-inflammatory agents. In this study, pseudane-VII dose-dependently diminished the production of NO and pro-inflammatory cytokines including IL-1β and IL-6 in LPS-activated macrophages (Figure 3 and Figure 5).

MAPKs are widely known serine, threonine, and tyrosine protein kinases and are important in regulating diverse cellular events such as cell proliferation, differentiation, and inflammation [28]. Previous studies have reported that MAPKs are strongly stimulated by LPS-activated TLR4 receptors, and their phosphorylation is involved in LPS-induced NO and pro-inflammatory cytokine production. Moreover, phosphorylated MAPKs trigger the phosphorylation of the NF-κB-p65 subunit, which is subsequently translocated into the nucleus where it regulates the transcription of inflammation-associated genes [29]. Therefore, we examined the effect of pseudane-VII on MAPK and NF-κB-p65 phosphorylation. Pseudane-VII treatment significantly decreased phosphorylation of ERK, JNK, p38 MAPK, and NF-κB-p65 in LPS-activated macrophages (Figure 6A,B). Moreover, for the first time, pseudane-VII was shown clearly to suppress the expression of iNOS and IL-1β on macrophage in LPS-stimulated mice (Figure 7). 

Taken together, the results demonstrate that pseudane-VII from *Pseudoalteromonas* sp. M2 metabolites suppressed NO synthesis and down-regulated pro-inflammatory cytokine production (IL-1β and IL-6) in an LPS-stimulated macrophage. Inhibition of NO and pro-inflammatory cytokine production was involved in the inhibition of MAPKs and NF-κB signaling in LPS-stimulated macrophages. The present study demonstrates that pseudane-VII has potential as a treatment for inflammatory responses and diseases.

## 4. Materials and Methods

### 4.1. Reagents

3-(4,5-Dimethyl-2-thiazolyl)-2,5-diphenyl-2H-tetrazolium bromide (MTT), Griess reagent, and TRI Reagent were purchased from Sigma Chemical Co. (St. Louis, MO, USA).

### 4.2. Extraction and Purification of Secondary Metabolites

Strain *Pseudoalteromonas* sp. M2 was inoculated into 4 L marine broth 2216 (MB; Difco, Franklin Lakes, NJ, USA) and incubated for 48 h at 22 °C with shaking. The strain culture was repeated 4 times, and cultivated 16 L totally.

To obtain secondary metabolites, the cell culture medium was centrifuged at 20,000× *g* for 30 min to remove precipitates. The supernatant was collected and treated with equal volume of ethyl acetate and shaken at 300 rpm for 15 min using JEIO TECH RS-1 recipro shaker (Jeio Tech, Daejeon, Korea). Then the ethyl acetate layer (upper layer) was vacuum-dried using Speed-Vac (Labconco, Kansas City, MO, USA) and extract was diluted in 50% methanol (*v*/*v* in deionized water). Each extract was purified by high-pressure liquid chromatography (HPLC) on a Waters AutoPurification System (Waters, Milford, MA, USA) with a QDa detector and a Waters X bridge prep C18 Column (19 × 250 mm, 5 μm) with a gradient of A (0.1% formic acid *v*/*v* in deionized water) and B (acetonitrile *v*/*v* in deionized water) at flow rate of 25 mL/min. The initial gradient composition (90% A/10% B) was held for 2.8 min, increased to 65% B in 43 min, and then decreased to 0% A in 45 min held for 5 min.

### 4.3. LC-MS Analysis of A5 (Pseudane VII)

LC/MS analyses were carried out using an LTQ Orbitrap XL (Thermo Electron Co., Waltham, MA, USA) coupled to an Accelar ultra-high pressure liquid chromatography system (Thermo Electron Co.). Chromatographic separation of metabolites was conducted using a ACQUITY UPLC^®^ BEH C_18_ column (2.1 × 150 mm, 1.7 μm), operated at 40 °C and using mobile phases A (water with 0.1% formic acid) and B (acetonitrile with 0.1% formic acid). The initial gradient composition (95% A/5% B) was held for 0.5 min, increased to 80% B in 10.0 min, decreased to 0% A in 10.1 min and held for 1.9 min. For recycling, the initial gradient composition was restored and allowed to equilibrate for 3.0 min. The LC-MS system consisted of heated electrospray ionization probe (HESI-II) as the ionization source. HESI was operated at 300 °C with spray voltage of 5.0 kV. The nebulizer sheath and auxiliary gas flow rates were set at 50 and 5 arb, respectively. MS analysis was performed with polarity switching, and the following parameters for MS/MS scan: *m*/*z* range of 150–1000; collision-induced dissociation energy of 45%; data-dependent scan mode. The Orbtrap analyzer was used for high-resolution mass spectra data acquisition with a mass resolving power of 30,000 FWHM at *m*/*z* 400.

### 4.4. Nuclear Magnetic Resonance (NMR) Analysis

^1^H and ^13^C NMR spectra were recorded on Bruker AvanceII 400 (Bruker, Billerica, MA, USA) in MeOD solutions. Working frequencies were 400.1 and 101.0 MHz for ^1^H and for ^13^C, respectively. 

### 4.5. Cell Culture and Pseudane-VII Treatment

The RAW 264.7 murine macrophage cell line was purchased from American Type Culture Collection (Manassas, VA, USA). RAW 264.7 cells were cultured at 37 °C with 5% CO_2_ in Dulbecco’s Modified Eagle’s Medium (DMEM) supplemented with 10% FBS, 200 IU/mL penicillin, 200 μg/mL streptomycin, 4 mM l-glutamine, and 1 mM sodium pyruvate (complete medium). Pseudane-VII was reconstituted in dimethyl sulfoxide (DMSO) at a concentration of 20 mM and then diluted to the desired concentration in DMEM (final DMSO concentration 0.1% *v*/*v*). In the control (untreated) samples, an equal concentration of DMSO was used.

### 4.6. Cytotoxicity Assay

Cytotoxicity was measured by colorimetric MTT assay. RAW 264.7 cells (2 × 10^4^ cells/well) in complete medium were seeded into a 96-well cell culture plate; various concentrations of pseudane-VII (0 to 100 μM) were added to the wells, and the plate was incubated in 37 °C for 24 h. After treatment, medium containing pseudane-VII was removed and MTT (0.5 mg/mL) solution was added to each well. After incubation in 37 °C for 4 h, MTT solution was removed and the formazan product was dissolved in solvent (1:1 = dimethyl sulfoxide:ethanol) resulting in a colored solution. Absorbance of the formazan solution was measured at 570 nm using an ELISA microplate reader (BioTek, Winooski, VT, USA).

### 4.7. NO Assay

RAW 264.7 cells (5 × 10^4^ cells/well) in complete medium were seeded into a 96-well culture plate. Cultured cells were pretreated with various concentrations of pseudane-VII (0 to 100 μM) for 2 h, and then incubated in the presence or absence of LPS for 22 h. After incubation, the culture medium was mixed with an equivalent volume of 1 × Griess Reagent and incubated for 15 min at room temperature. Absorbance was measured at 450 nm using an ELISA microplate reader.

### 4.8. Reverse Transcription (RT)-PCR

RAW 264.7 cells (1 × 10^6^ cells/well) in complete medium were seeded into a 6-well culture plate. Cultured cells were pretreated with various concentrations of pseudane-VII (0 to 100 μM) for 2 h, and then cells were incubated for 6 h in the absence or presence of LPS. After incubation, pseudane-VII-treated cells were collected by centrifugation and total RNA was isolated from the cells using TRI Reagent according to manufacturer’s protocol. To synthesize cDNA, 0.5 μg of total RNA was primed with oligo dT and reverse transcribed using a mixture of M-MLV RTase, dNTP, and reaction buffer (Promega, Madison, WI, USA). To measure the mRNA level of inflammatory factors including *inos*, *cox-2*, *il-1β*, *il-6* and *tnf-α*, we designed the primers for target genes (Bioneer, Daejeon, Korea). cDNA was amplified using the Gene Atlas G02 gradient thermal cycler system (Astec; Fukuoka, Japan) e-Taq DNA Polymerase kit (Solgent, Daejeon, Korea) and the primers. The PCR products were visualized by fluorescent dye and a UV transilluminator.

### 4.9. Western Blot Analysis

Cells were pretreated in the absence or presence of 100 μM of pseudane-VII then exposed to LPS (200 ng/mL). Following 15, 30, 45 min, and 24 h of incubation at 37 °C, cells were washed twice with cold PBS and lysed with modified RIPA buffer (150 mM sodium chloride, 1% Triton X-100, 0.5% sodium deoxycholate, 0.1% sodium dodecyl sulfate (SDS), 50 mM Tris (pH 8.0), 1 mM phenylmethylsulfonyl fluoride (PMSF), 2 μg/mL leupeptin, 1 μg/mL pepstatin, 1 mM sodium orthovanadate, and 100 mM sodium fluoride) for 30 min at 4 °C. Lysates were cleared by centrifuging at 14,000× *g* for 15 min at 4 °C. The protein content of cell lysates was determined using the Micro BCA assay kit (Pierce, Rockford, IL, USA) according to the manufacturer’s instructions. Equivalent amounts of protein were separated by 10% SDS-polyacrylamide gel electrophoresis (SDS-PAGE) and electrophoretically transferred to a polyvinylidene difluoride (PVDF) membrane. The membrane was placed into a blocking solution (5% nonfat milk) at room temperature for 1 h. After blocking, blots were incubated with anti-iNOS, anti-COX-2, anti-ERK1/2, anti-phospho-ERK1/2 (p-ERK), anti-p38, and phospho-p38 (p-p38), antibodies overnight (Santa Cruz Biotechnology, Dallas, TX, USA). Horseradish peroxidase-conjugated anti-rabbit and anti-mouse antibodies (Santa Cruze Biotechnology) were used as the secondary antibodies. Band detection was performed using the enhanced chemiluminescence (ECL) detection system and exposed to radiographic film. Pre-stained blue markers were used for molecular weight determination.

### 4.10. Enzyme-Linked Immunosorbent Assay (ELISA)

RAW 264.7 cells (5 × 10^4^ cells/well) were seeded into a 96-well culture plate. Cells were pretreated with various concentrations of pseudane-VII for 2 h, and then incubated in the absence or presence of LPS for 22 h. IL-1β, IL-6 and TNF-α released into the culture supernatants was measured using Mouse IL-1β, IL-6 and TNF-α ELISA MAX™ Deluxe Sets (BioLegend; San Diego, CA, USA), according to manufacturer’s protocol. Briefly, standards and samples were incubated on a capture antibody-coated plate at 4 °C overnight. Detection antibody was incubated for 1 h and Avidin-HRP was added to bind to the detection antibody. Following the addition of substrate solution to each well, the reaction was stopped by stop solution (2N H_2_SO_4_). Absorbance was measured at 405 nm using an ELISA microplate reader.

### 4.11. Isolation of IL-1β Producing-Macrophages from Spleen

Mice were injected intraperitoneally (i.p.) with Pseudane-VII (1 mg/kg) every 3 days before the administration of 1 mg/kg of LPS in a lateral vein of the tail. Twenty-four hours after the LPS challenge, they were killed, their spleens were disrupted and the cells were centrifuged with 400× *g* for 5 min, resuspended in DMEM media. The cells were fixed and permeated with the cytofix/cytoperm kit according to manufacturer’s instructions. Intracellular IL-1β was stained with fluorescein R-phycoerythrin (PE)-conjugated antibody in permeation buffer. The cells were gated on F480-FITC positive cells for macrophages. The stained cells were analyzed using a flow cytometry (Beckman, FC500) and Kaluza software.

### 4.12. Statistical Analysis

The data were analyzed by one-way analysis of variance (ANOVA) followed by Scheffe’s post-hoc test using SPSS (IBM; Armonk, NY, USA). The results are presented as the mean ± standard deviation. The differences were considered statistically significant at *p* < 0.01.

## Figures and Tables

**Table 1:** The NMR data of A5 (Pseudane VII), recorded at ^1^H-400 MHz; ^13^C-100 MHz in Methanol-*d*_4_.

No. C/H	δC ppm	δH (ppm), Integration, Multiplicity, *J* (Hz)
1(CH)	123.6	7.33 (1H, t, *J* = 8.05, 7.61 Hz)
2(CH)	131.8	7.58 (1H, t, *J* = 7.96, 7.61 Hz)
3(CH)	118.3	7.71 (1H, d, *J* = 7.96 Hz)
4(C)	140.5	-
5(C)	125.0	-
6(CH)	125.4	8.36 (1H, d, *J* = 8.05 Hz)
8(C)	154.9	-
9(CH)	108.3	6.24 (1H, s)
10(C)	178.9	-
11(CH_2_)	34.4	2.68 (2H, t, *J* = 7.50 Hz)
12(CH_2_)	28.5	1.72 (2H, tt, *J* = 7.50, 6.60 Hz)
13(CH_2_)	29.5	1.28 (2H, tt, *J* = 7.54, 6.60 Hz)
14(CH_2_)	28.7	1.10 (2H, quin, *J* = 7.54, 7.40 Hz)
16(CH_2_)	30.8	1.19 (2H, quin, *J* = 7.40 Hz)
17(CH_2_)	21.9	1.28 (2H, tt, *J* = 7.54, 6.60 Hz)
18(CH_3_)	13.8	0.87 (3H, t, *J* = 6.77 Hz)
